# Transcriptomic meta-analysis reveals up-regulation of gene expression functional in osteoclast differentiation in human septic shock

**DOI:** 10.1371/journal.pone.0171689

**Published:** 2017-02-15

**Authors:** Samanwoy Mukhopadhyay, Pravat K. Thatoi, Abhay D. Pandey, Bidyut K. Das, Balachandran Ravindran, Samsiddhi Bhattacharjee, Saroj K. Mohapatra

**Affiliations:** 1 National Institute of Biomedical Genomics, Kalyani, West Bengal, India; 2 Department of Medicine, Sriram Chandra Bhanja Medical College and Hospital, Cuttack, Odisha, India; 3 Institute of Life Sciences, NALCO Square, Bhubaneswar, Odisha, India; Charles P. Darby Children’s Research Institute, 173 Ashley Avenue, Charleston, SC 29425, USA, UNITED STATES

## Abstract

Septic shock is a major medical problem with high morbidity and mortality and incompletely understood biology. Integration of multiple data sets into a single analysis framework empowers discovery of new knowledge about the condition that may have been missed by individual analysis of each of these datasets. Electronic search was performed on medical literature and gene expression databases for selection of transcriptomic studies done in circulating leukocytes from human subjects suffering from septic shock. Gene-level meta-analysis was conducted on the six selected studies to identify the genes consistently differentially expressed in septic shock. This was followed by pathway-level analysis using three different algorithms (ORA, GSEA, SPIA). The identified up-regulated pathway, Osteoclast differentiation pathway (hsa04380) was validated in two independent cohorts. Of the pathway, 25 key genes were selected that serve as an expression signature of Septic Shock.

## Introduction

Septic shock (SS) is a serious medical condition that claims many lives every year worldwide. Approximately 2% of the patients admitted to the hospital are diagnosed with SS [1], with mortality of 40–60% within 30 days [2]. Of these patients, half are treated in the intensive care unit (ICU), representing 10% of all ICU admissions [2, 3]. The number of cases in USA exceeds 750,000 per year [2], and has been estimated up to 19 million cases worldwide per year [4]. Incomplete grasp of SS biology is compounded by the lack of specific drug for treating the condition. With a number of unsuccessful clinical trials, there is urgent need for new directions in research [5].

Genome-wide expression profiling offers a detailed picture of the condition and enables identification of genes and pathways of diagnostic, prognostic or therapeutic relevance [6]. There have been a number of studies investigating gene expression in sepsis and septic shock leading to very interesting discoveries, such as altered zinc metabolism in sepsis [7], and clinically relevant grouping of cases of septic shock [8]. The common goal of these previous analyses was to detect interesting genes associated with sepsis or septic shock. Gene-level analysis is inherently not geared toward detection of pathways, which are sometimes modulated even in the absence of significant gene-level changes in expression.

The purpose of this study was to investigate genome-wide host response to SS by combining the power of multiple studies and using complementary bioinformatics methods. Analysis was performed at two levels: genes and gene sets. A “gene set” or pathway consists of a set of functionally related genes, and provides higher-order information about gene expression and valuable insights into the biology of a disease. Accordingly, we have laid emphasis on robust discovery of pathway(s) differentially expressed in SS.

## Materials and methods

### Search strategy and selection criteria

We searched the popular online database PubMed with the search string (“Systemic Inflammatory Response Syndrome”[MeSH] OR “septic shock”[MeSH] OR “Shock, Septic”[MeSH] OR “Endotoxemia”[MeSH]) AND (“gene expression profiling”[MeSH] OR “transcriptome”[MeSH] OR “microarray analysis”[MeSH] OR “Oligonucleotide Array Sequence Analysis”[MeSH]) with “Human” filter. Additionally, we searched the following gene expression databases: (1) National Centre for Biotechnology Information Gene Expression Omnibus (GEO) and (2) European Bioinformatics Institute ArrayExpress.

**Fig 1:**
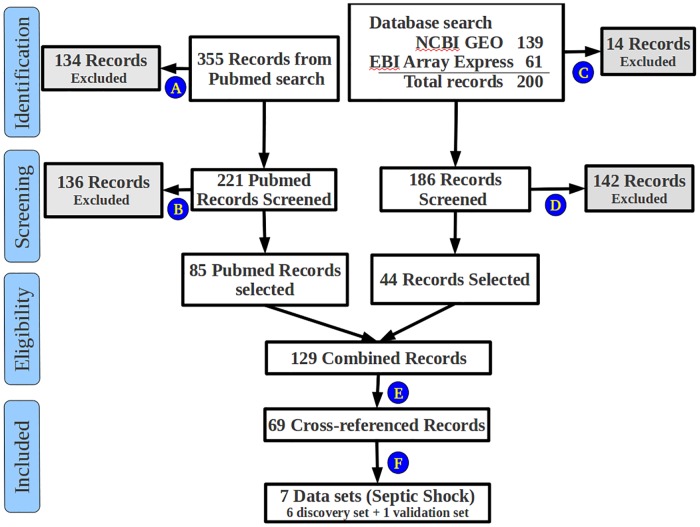
PRISMA Flow Chart. A flow chart of electronic search results made across gene expression databases. All records with the following attributes were excluded: A: Non-human Genome; Genotyping study, not gene expression; Review article; Comment article; B: In vitro study; microRNA profiling; Assay for bacterial identification; eQTL studies; Data analysis methods; Full text article not available; C: Non-human Genome; Genotyping study; No gene expression data available; D: In vitro study; microRNA profiling; Assay for bacterial identification; eQTL studies; Data not available; E: Viral infection; Different tissue than blood; Duplicate studies removed; F: Not septic shock; Source of RNA is specific cell type not whole blood.

All queries were made on the 3rd January 2017. Entries from the gene expression databases were cross-referenced with publications retrieved from PubMed. Selection of studies was based on the organism (human subjects), tissue of origin (circulating leukocytes from whole blood samples) and the platform technology (gene expression microarray) Fig 1. Only data sets published as full reports were selected.

All records with the following attributes were excluded: non-human genome; microRNA profiling; genotyping study, not expression; eQTL study; analysis methods; source of RNA is tissue other than blood; source of RNA is specific cell type, not whole blood; in vitro study; assay for bacterial identification; viral infection; not septic shock; review article; comment article; full text not available. The exclusion criteria employed at different levels are described in the legend of Fig 1.

The selected records were divided into two data sets: (a) Discovery set with six studies from the same laboratory (Table 1) and (b) Validation set (Table 2) from a different laboratory (GSE57065).

**Table 1:** The table shows characteristics (such as sample size, study design, clinical parameters for inclusion criteria and details about the platform technology used to generate the data) of the six selected gene expression studies of septic shock (SS). Each row represents one study.

Study ID	Author-Year	PMID	Study Design	No. of Samples	Mean Age (yr)	Clinical Setting	Inclusion Criteria	Control Group	Tissue	Sample collection and RNA isolation method	Platform	Data normalization
GSE4607	Wong2007 Cvijanovich2008 Wong2012	17374846 18460642 23107287	Observational study	84	Children <10 years of age	PICU	Septic Shock	Healthy controls	Whole blood	PAXgene	Affymetrix Human Genome U133 Plus 2.0 Array	RMA
GSE8121	Shanley2007	17932561	Longitudinal	75	Children <10 years of age	PICU	Septic Shock	Healthy controls	Whole blood	PAXgene	Affymetrix Human Genome U133 Plus 2.0 Array	RMA
GSE9692	Cvijanovich2008	18460642	Retrospective observational study	45	Children <10 years of age	PICU	Septic Shock	Healthy controls	Whole blood	PAXgene	Affymetrix Human Genome U133 Plus 2.0 Array	RMA
GSE13904	Wong2009	19325468	Prospective observational study	124	Children <10 years of age	Paediatric wards	Septic Shock	Healthy controls	Whole blood	PAXgene	Affymetrix Human Genome U133 Plus 2.0 Array	RMA
GSE26378	Wynn2011	21738952	Prospective observational study	103	Children <10 years of age	Paediatric wards	Septic Shock	Healthy controls	Whole blood	PAXgene	Affymetrix Human Genome U133 Plus 2.0 Array	RMA
GSE26440	Wong2009	19624809	Prospective observational study	130	Children <10 years of age	Paediatric wards	Septic Shock	Healthy controls	Whole blood	PAXgene	Affymetrix Human Genome U133 Plus 2.0 Array	RMA

**Table 2:** The table shows characteristics (such as sample size, study design, clinical parameters for inclusion criteria and details about the platform technology used to generate the data) of the single selected gene expression study of septic shock (SS). Each row represents one study.

Study ID	Author-Year	PMID	Study Design	Total No. of Samples	Mean Age (yr)	Clinical Setting	Inclusion Criteria	Control Group	Tissue	Sample collection and RNA isolation method	Platform	Data normalization
GSE57065	Monneret2014	26215705	Observational study	107	Adult	ICU	Septic Shock	Healthy controls	Whole blood	PAXgene	Affymetrix Human Genome U133 Plus 2.0 Array	RMA

### Pre-processing of data

Normalized gene expression data from the series matrix files were transformed to logarithmic scale (base 2). Expression intensity for each entrez gene ID was calculated after removing duplicated probe sets. Genes common to all studies were included in the analysis. Redundant samples (other than control and SS) were excluded from analysis.

### Identification of differentially expressed genes

For all genes within each study, we applied a Welch’s 2-sample t-test [9] to compare expression values in samples of SS with that in samples of control subjects. A single overall p-value was then computed for each gene by meta-analyzing individual study level t-statistics, as described below.

We first transformed each individual t-statistic to a Z-score Z_*i*_ retaining its sign by using the quantile transformation:

$$\begin{array}{c} Z_{i}=\phi^{-1}\left(F_{t_{n_{i}^{SS}+n_{i}^{CON}-2}}\left(t_{i}\right)\right) \end{array}$$ (1)

We then followed a fixed effects meta-analysis [10] approach, where the Z-scores are combined using an optimal linear combination with weights equal to square root of the effective sample size of each study (i.e., the harmonic mean of case and control sample sizes). Since some of the studies have overlapping cases and controls as shown in S1 Table, we derived the correlation matrix among the Z-scores of the 6 studies using a previously known correlation formula for case-control studies [11]. The variance of the linear combination is thus derived as the sum of variances of the numerator terms added to their pairwise covariances based on the inter-study correlations R_*ij*_. This gave the combined meta-analyzed Z-score Z_*meta*_:

$$\begin{array}{c} Z_{meta}=\frac{\sum_{i}\sqrt{n_{i}^{eff}}Z_{i}}{\sum_{i}n_{i}^{eff}+\sum_{j\neq i}\sqrt{n_{i}^{eff}}\sqrt{n_{j}^{eff}}R_{ij}};\;\;\;\;\;\;n_{i}^{eff}=\frac{2\;n_{i}^{SS}\times n_{i}^{CON}}{n_{i}^{SS}+n_{i}^{CON}} \end{array}$$ (2)

The final overall 2-sided p-value *p*_*meta*_ was obtained as,

$$\begin{array}{c} p_{meta}=2\times \Phi (1-|Z_{meta}\left|\right), \end{array}$$ (3)

where Φ denotes the standard normal distribution function.

### Over-Representation Analysis (ORA)

The set of up-regulated genes was then subjected to over-representation analysis by applying hypergeometric test [12]. A significant association was detected at threshold of p < 0.001.

### Gene Set Enrichment Analysis (GSEA)

Permutation test was performed by randomly scrambling the sample labels (case/control), producing the corresponding ranked list, and computing the enrichment score (ES) of the gene set for this permuted data set [12]. Pathways with observed ES very different (p = 0.0) from the null distribution were considered significant. For each pathway a meta-analyzed GSEA p-value was obtained using Fisher’s p-value product method [13].

### Signaling Pathway Impact Analysis (SPIA)

By combining two different evidences (one from the analysis using the hypergeometric model and the other from the probability of perturbation that takes the pathway topology into account), a pathway-level score was computed. For each pathway, Fisher product of the six p-values (one for each study) was calculated to return one p-value for pathway. The ten most significantly perturbed pathways were considered.

### NIBMG validation cohort

Blood samples were collected from the patients of SS and healthy subjects after obtaining approval from the Institutional Ethical Committees of the National Institute of Biomedical Genomics, Kalyani, India and SCB Medical College Hospital, Cuttack, India. All the methods were carried out in accordance with the approved guidelines. Informed written consent was obtained from all subjects who participated in the study. 12 healthy control and 9 cases of SS were included in the study.

Genes of the Osteoclast differentiation pathway were extracted and mean gene expression computed. Scatter plot of SS versus control was generated for visual inspection of the data. P-value for up-regulation of the pathway was computed using a permutation test. Further, significant genes of the pathway were selected based on two filters: FDR p-value < 0.05 and high expression intensity (100 or more).

### Sample preparation and data processing

Whole blood was collected from the subjects in PAXgene Blood RNA tubes (BD/Preanalytics) and incubated at ambient temperature for at least 2 hours. RNA was isolated using PAXgene Blood RNA kit (Qiagen) as per manufacturer’s instructions. Total RNA yield and quality were checked in Nanodrop 2000 spectrophotometer and Agilent Bioanalyzer RNA Nano 6000 chip. RNA samples with a RIN number ≥ 6 were converted to cRNA and hybridised onto either HumanHT-12 v4 BeadChip (Illumina) or Human Gene 2.0 ST Arrays (Affymetrix). Gene expression intensities were read using the standard protocol provided by the manufacturer.

Raw data files from the platform HumanHT-12 v4 BeadChip (Illumina) were read with the help of package “limma” [14] followed by background correction and normalization using the control probes. For the samples processed on Human Gene 2.0 ST Arrays (Affymetrix), RMA-normalization was performed. Each probe ID was mapped to its corresponding Entrez gene ID from the gene symbol by the help of “org.Hs.eg.db” package. In case of duplicated/multiple probes for a single Entrez gene id, the probe with the highest variance was retained using the package “genefilter”). Correction of batch effect was performed with the *ComBat* function of “sva” [15] package.

Genes of the Osteoclast differentiation pathway were extracted and mean gene expression computed. Scatter plot of SS versus control was generated for visual inspection of the data. P-value for up-regulation of the pathway was computed using a permutation test. Further, significant genes of the pathway were selected based on two filters: FDR [16] p-value < 0.05 and high expression intensity (100 or more).

### Permutation test for enrichment

Permutation-based enrichment test was performed to provide evidence for overall up-regulation of Osteoclast differentiation pathway in SS (NIBMG validation cohort). For this, we used the package “resample” [17] to calculate the permutation-based p-value accounting for correlation among the pathway genes. First, we calculated the proportion of significantly up-regulated (p < 0.05) genes in the pathway by using a two-sample t-test to test for up-regulation of each pathway gene. This was observed to be 0.447. Next, we reshuffled the sample groups (i.e. case control status) 100000 times and similarly calculated the proportion of up-regulated genes for each permutation replicate. Finally, the permutation-based p-value was obtained as the proportion of replicates where the simulated proportion was greater than the observed value.

### GSE57065 validation data set

Normalized data for the study GSE57065 were retrieved from NCBI GEO database and the expression matrix for the selected gens was extracted. Principal Components Analysis was performed and 3D plot of the data generated to demonstrate clear separation of SS and control samples.

### Topology analysis of the selected genes

The genes were divided in to three groups based on location within the KEGG pathway hsa04380 (Osteoclast Differentiation): membrane, nucleus or intermediate. For each group, log-fold changes in gene expression were displayed as box plot. KEGG pathway hsa04380 map was color-coded following that in the box plot.

### Expression difference between outcome groups

Survival information for the subjects was derived from the phenotype information provided under the studies. Expression data for each of the selected genes were displayed in the form of box plots. Welch’s 2-sample t-test [9] was performed between survivors and non-survivors.

### Availability of data and materials

The dataset(s) and the R code supporting the conclusions of this article are available under the project **ssnibmg** in the repository https://figshare.com.

## Results

### Selection of studies and meta-analysis

**Fig 2:**
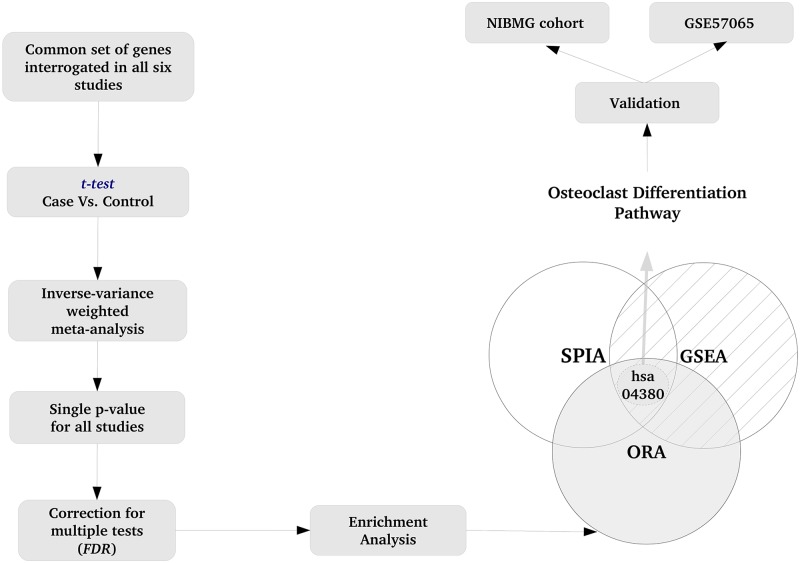
Overview of the analysis work-flow. First, gene-level analysis was performed by comparing SS with control. The p-values from multiple studies were combined to compute a single p-value per gene. The p-values were adjusted for multiple testing (FDR). 200 genes with low p-value (p < 0.001) and large fold change (2 or greater) were considered significantly differentially expressed (up-regulated) in SS. The 200 up-regulated genes were subjected to Over-Representation Analysis (ORA). Additional analyses [Gene Set Enrichment Analysis (GSEA) and Signaling Pathway Impact Analysis (SPIA)] were carried out on the genome-wide expression data. A single pathway (hsa04380—Osteoclast Differentiation) was returned as the common result of the three analyses. Up-regulated gene expression of Osteoclast differentiation pathway in SS was validated in two independent cohorts.

By systematic search of literature, NCBI GEO and other gene expression databases, we selected six studies of SS (Table 1). Normalized gene expression data of the studies were retrieved from GEO and analyzed to detect differentially expressed genes (Fig 2). Differential expression was measured in terms of both log-fold change and p-value. For each gene, SS was compared with control and the six p-values were combined to generate a single p-value per gene. For each gene, the six log-fold changes were averaged to produce a single log-fold change. Using stringent criteria (adjusted p-value < 0.01, fold change of 2 or more), we discovered 200 genes that were consistently up-regulated in SS. We did not discover any gene that was down-regulated at this level of significance, i.e., p < 0.01 and fold change of half or less (S1 Fig).

### The 200 genes up-regulated in SS

These included genes associated with inflammation and innate immunity, critical illness, anti-microbial activity, complement and coagulation systems, carbohydrate and lipid metabolism, and others. This is consistent with other reports including those by the authors of the primary data sets (S2 Table). While many of these systems are known to be involved in SS, there were some surprises, such as, up-regulation of genes of bone metabolism. With strong evidence in favour of altered expression at gene level, we proceeded to analyse the data at pathway (gene set) level.

#### Pathway analysis: ORA, GSEA, SPIA, IPA

A pathway is a set of genes that participate in a single functional module. When a large number of genes in the same pathway are perturbed (or up-regulated, in this case), then the pathway itself may be inferred to be perturbed. Secondly, small changes in gene expression are usually not detectable with standard methods. However, if the changes happen in a co-ordinated fashion over a set of functionally related genes (i.e., a pathway), then the cumulative effect of the small gene-level changes shall result in a large and detectable perturbation at the pathway-level. Lastly, the gene set in a pathway has a specific network structure or topology, such that, the nodes proximal to the point of origin (for example, the signal from the membrane), has a greater regulatory impact compared to the distal nodes. All these considerations were taken into account in the current analysis.

Kyoto Encyclopedia of Genes and Genomes (KEGG) [18] is a database of gene sets (pathways) where each pathway consists of multiple genes and a gene may belong to more than one pathway. We sought to find the pathway(s) that is (are) significantly “enriched” with the genes in our list of 200. Using the method of “Over-Representation Analysis” (ORA), three pathways were identified: hsa04610—Complement and Coagulation Cascades, hsa04380—Osteoclast Differentiation, and hsa05202 -Transcriptional Misregulation in Cancer (S3 Table). The complement system, when activated, leads to recruitment of inflammatory cells and elimination of pathogens, and has been implicated in SS [19]. Similarly, blood coagulation, although an essential defense mechanism for blocking off the pathogen and inflammatory spread in the initial phase, can be detrimental in later stages of SS, in the form of disseminated intravascular coagulation (DIC) [20]. The other two pathways are not commonly associated with SS.

Subsequently, we applied two other tools that are methodologically independent of ORA, and provide additional support to our finding (Fig 2). Gene Set Enrichment Analysis (GSEA), using a global i.e., genome-wide, search strategy, detected the KEGG pathway (s) with significant up-regulation in SS compared to control. Signaling Pathway Impact Analysis (SPIA), combined elements of ORA and GSEA, with attention to gene-gene interactions and pathway topology. There was a single pathway (hsa04380: Osteoclast differentiation) that was common to the results from the three approaches (ORA, GSEA, SPIA). Further, we performed Ingenuity Pathway Analysis (IPA) with the top 200 up-regulated genes (found in discovery cohort), that returned a list of pathways, including those related to bone loss, p38 MAPK signaling, complement system and TLR signaling. The network with significant biological function termed “infectious disease, inflammatory response, connective tissue disorders” was discovered to be differentially regulated in SS (S2 Fig). Over all, IPA results were similar to the pathway analysis with KEGG, and generally consistent with the finding of altered skeletal function in SS. Thus, multiple analytic approaches, ORA, GSEA, SPIA and IPA, together revealed hsa04380 (Osteoclast Differentiation) with unequivocal transcriptional up-regulation in SS. We then sought to validate the pathway up-regulation in independent data sets.

### Validation of osteoclast differentiation pathway (hsa04380)

**Fig 3:**
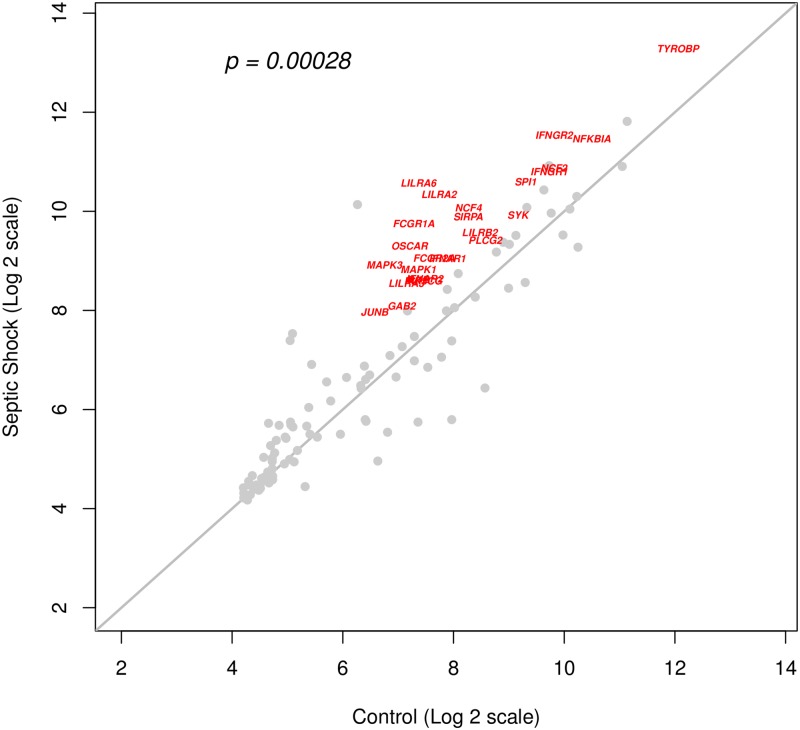
Scatter plot of Osteoclast differentiation pathway genes from expression data generated in our laboratory (NIBMG cohort) validation cohort. Each point corresponds to a single gene, and its coordinates reflect mean gene expression in healthy controls (x-axis) and SS (y-axis). The points close to the identity line (the diagonal in the figure), correspond to genes with similar expression level in the control and the SS groups. Points away from the diagonal represent genes that are differentially expressed in SS (up-regulated if the points are in the upper half of the plot area; down-regulated otherwise). Many of the genes are up-regulated in SS as shown by a large number of points deviated from the diagonal toward the SS-axis. The up-regulation of these genes in SS is statistically significant (p = 0.00028, permutation test for enrichment). Additional testing was performed for each gene (unpaired t-test between control and SS) leading to selection of individual genes in the pathway that were significantly up-regulated after a correction of multiple-testing at an FDR level of 0.05. An expression filter was applied to identify genes that showed a high fold-change (2 or more) and were expressed in significant amounts (intensity of 100 or more). These genes have been shown in red on the plot. It may be noted that there are a few genes that are down-regulated in SS, represented by points below the diagonal line. However, permutation test clearly revealed up-regulation of the pathway (p = 0.00028) over-riding a few individual gene-level effects.

**Fig 4:**
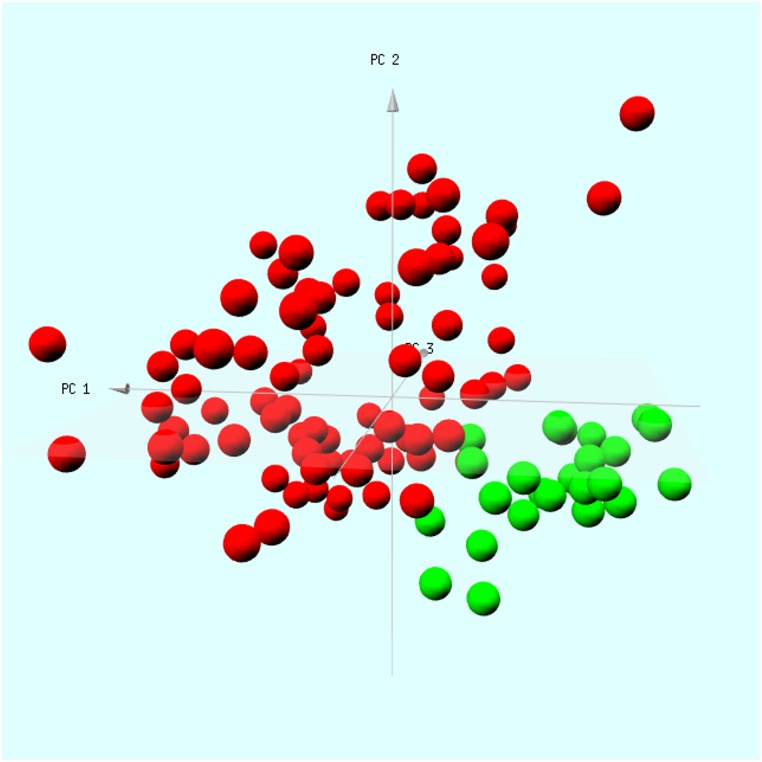
Principal components Analysis on data extracted from an independent Validation dataset GSE57065 that was not used in the discovery phase. Each point corresponds to fold-change in expression of 25 genes for a single individual. Color of each point denotes the disease status: red for SS, greeen for healthy controls. Using the selected 25 genes of the pathway, this plot clearly shows separation between healthy control and SS.

Validation was performed in two stages, first using data generated by our laboratory, followed by further testing on an independent data set. Batch-corrected whole blood gene expression data (S3 Fig) of the Osteoclast differentiation pathway was obtained by our laboratory from an independent set of human subjects (Table 3). Mean expression values (for the two groups: control and SS) were calculated and a scatter-plot generated (Fig 3). In this plot, each point corresponds to a single gene. The points near the identity line (the diagonal in the figure), correspond to genes with similar expression level in control and SS groups. The genes that are up-regulated in SS are expected to be significantly deviated from the diagonal toward the SS axis. Indeed, for most of the genes, there is much higher expression in SS, as shown in Fig 3. Additional evidence for up-regulation of the Osteoclast differentiation pathway was obtained from the permutation-based enrichment test (permutation p = 0.00028; 100000 replicates). On examining at the gene-level, we observed that 60 genes were FDR significant and 25 genes passed the expression filters. S4 Fig shows the box plots of these highly significant genes in the validation cohort from our laboratory. These 25 genes were significantly up-regulated in both the Discovery set (6 studies) and the NIBMG validation set. Next, we sought to validate the expression of the selected genes in a new data set (GSE57065) that was not used in the discovery phase. Majority of the genes (22 out of 25 genes, FDR < 0.01) were significantly up-regulated in SS. Principal Components Analysis showed clear segregation of SS from control samples (Fig 4).

**Table 3:** Clinical characteristics of SS patients in Validation Cohort.

ID	Age	Sex	Primary Diagnosis	Vasopressor	Infective focus	APACHE II score	Pathogen Detected	Co-morbidities
5	70	M	Septic Shock	Dopamine	Left lower lobe pneumonia	20	*E. coli* (Sputum)	COPD
6	55	F	Septic Shock	Dopamine	Right lower lobe pneumonia	16	*Pneumococcus*	NA
19	14	F	Septic Shock	Noradrenaline	Right lower lobe pneumonia	16	NA	SLE
21	20	M	Septic Shock	Dopamine	Left lower lobe pneumonia	13	*Pneumococcus* (Sputum)	Alcoholism
34	32	M	Septic Shock	Dopamine	Right lower lobe pneumonia	10	*Pneumococcus* (Sputum)	Mitral Stenosis
50	38	M	Septic Shock	Noradrenaline	Right leg abscess	12	*S. aureus* (Pus)	Filariasis
53	75	M	Septic Shock	Dopamine	Right lower lobe pneumonia	38	*E. coli* (Sputum)	NA
54	38	F	Septic Shock	Noradrenaline	Right lower lobe pneumonia	26	*Pneumococcus* (Sputum)	NA
56	63	F	Septic Shock	Dopamine	Urinary Tract Infection	24	*E. coli* (Urine)	SLE

### Topology analysis of the significant genes

For the three topological gene groups, log-fold changes in expression for the Membrane-associated genes were observed to be higher than that for the Intermediate group, which was also lesser than that for the Nucleus group, consisting of two transcription factors (S5 Fig).

### Association with survival

We investigated if the selected genes are associated with outcome of the samples. Using available survival information from the studies, we observed that 13 of the 25 genes were over-expressed in survivors compared to non-survivors, at a significance level of p < 0.1 (S6 Fig).

## Discussion

Genome-scale analysis of multiple datasets, when performed at the level of pathways, is a powerful approach to discovery of functional modules that are potentially missed by gene-level testing. There exist several approaches to pathway analysis, such as ORA, GSEA and SPIA, each with its own advantages [21]. ORA considers the number of genes differentially expressed, but ignores any value associated (such as fold-change). GSEA considers all genes in a pathway and is more likely to detect coordinated changes in gene expression. SPIA, in addition, takes the topology of the pathway into account. Here, combination of three different methods gives leverage in finding the pathway that is unequivocally altered in SS.

In order to discover the stable blood transcriptional changes in SS, we conducted three different analyses (ORA, GSEA, SPIA) and intersected the resulting lists to obtain a single KEGG pathway (hsa04380—Osteoclast Differentiation Pathway). We confirmed this result in two validation cohorts and demonstrated that up-regulation of the pathway is distinct in SS compared to controls. To our knowledge, this is the first time that osteoclast differentiation pathway has been shown to be significantly perturbed in SS.

Osteoclasts are specialised bone cells of monocyte-macrophage lineage [22] responsible for bone resorption whereas osteoblasts are responsible for bone formation. It was earlier shown that there is increased bone resorption in critically ill patients [23]. However, our finding suggests that the role of this pathway may be more specific in SS.

**Fig 5:**
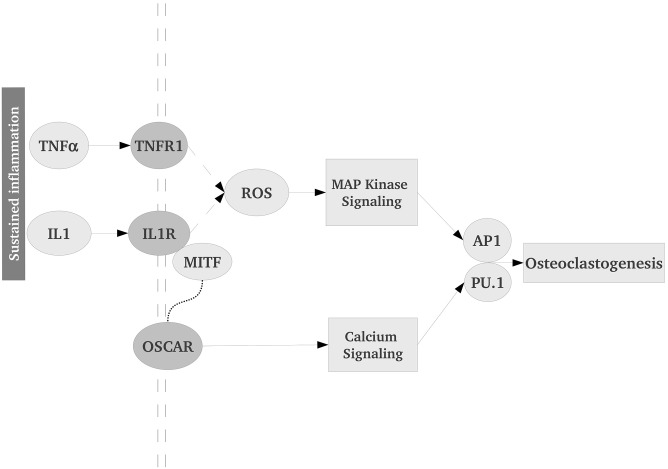
A simplified model of the Osteoclast Differentiation Pathway up-regulation in SS. Sustained inflammation leads to activation of osteoclast differentiation through proinflammatory cytokines TNF*α* and IL1, followed by MAP Kinase and Calcium signaling. Reactive Oxygen Species (ROS) play a major role in activation of MAP Kinase. OSCAR is an important receptor on the membrane that causes signal transduction via calcium-signaling. AP1 and PU.1, two important transcription factors, integrate multiple signals and induce expression of osteoclastogenesis-specific genes.

Of the pathway, we identified 25 key sigificant(*p* < 0.05) genes of this pathway (S4 Table). The genes whose encoded proteins form a signal transduction cascade from the plasma membrane to the nucleus. Based on these 25 genes, a simple model of osteoclast differentiation in SS is proposed (Fig 5).

In this model, the Osteoclast differentiation pathway may be conceptualised in terms of interactions among a few key processes or subnetworks. Firstly, pro-inflammatory cytokine-receptor complexes (such as, TNF*α*-TNFR and IL1*β*-IL1R) cause increased activity of the MAP Kinase pathway (with some role of reactive oxygen species) resulting in induction of AP1. Secondly, IL-1 and TNF*α* activate NF*κ*B to up-regulate expression of osteoclast specific genes including OSCAR (osteoclast associated receptor). The association of TNF*α*, IL-1, IL-6 and IL-7 has been detected in a variety of chronic inflammatory bone diseases and are significantly up-regulated in SS. Signal from OSCAR is transduced through intracellular calcium release, that is known to activate transcription factors, PU.1 (a well-known biomarker for osteoclast differentiation) [24] and AP1 (JUN, FOSL) [25]. Both PU.1 and AP1 are significantly up-regulated in SS. Mitochondrial dysfunction in SS causes generation of various reactive oxygen (and nitrogen) species, contributing to activation of MAP kinase pathway and subsequent osteoclastogenesis [26]. Although little attention has been paid to the role of ROS in differentiation of macrophages and monocytes into osteoclasts, we observed that MAP kinase pathway and genes associated with ROS generation are significantly up-regulated in SS.

Since our analysis was performed on whole blood gene expression data, we were interested in the cellular and plasma distribution of the gene products. A literature search for tissue specific expression revealed that many of these genes are expressed in neutrophils and monocytes, two important leukocyte sub-types. Additionally, almost all of the gene products have been reported to be detectable in plasma (S4 Table).

In most studies, discharge from the ICU is considered the survival end-point. However, the survivors themselves are prone to many long term complications including musculoskeletal ones. By rigorous analysis, we show that patients of SS have increased osteoclast differentiation. Since excessive osteoclast activity contributes to enhanced bone resorption, patients of SS (especially those that survive the acute episode) are likely at risk for bone loss and fracture. Therefore, this pathway is a potential target for therapeutic intervention.

We found interesting patterns in the topological characteristics of 25 genes selected. Genes associated with the membrane had a higher differential expression compared with controls and also higher expression (although at p < 0.1) in survivors compared to non-survivors. Further exploration in this direction is needed to understand the clinical implications. Lastly, although our study was conducted on mRNA abundance in whold blood, the 25 selected gene products are also detectable in peripheral circulation (S4 Table).

It is to be noted that we did not check for ‘publication bias’ as this kind of bias is generally meaningful for candidate gene studies, as opposed to omics studies. (It is highly unlikely for a transcriptomic study to not detect any Differentially Expressed gene and remain unpublished). Additionally, had we tried to pool the transcriptomic data, many potential biases would creep in. Therefore, we meta-analyzed the summary t-statistics, allowing us to avoid such biases. Of note, the data within each study was already available as normalized data to remove intra-study biases. While it is still theoretically possible for some genes to behave heterogeneously between studies, it would be biologically rare to occur within the same tissue (whole blood in this case). All the studies chosen had most subjects of Caucasian ethnicity making inter-study heterogeneity in gene-expression less likely to appear. We chose our initial phenotype (Septic Shock) to be as homogenous as possible. This reduced our number of studies for our meta-analysis to only 7. Hence accounting for inter-study heterogeneous behavior of genes (e.g. random effects models) would reduce our statistical power without significant benefits.

This study has a limitation in terms of heterogeneous phenotypes between the discovery and validation cohorts, such as age, and organ involvement. SS is a severe form of disease where the multiorgan involvment and host response is usually similar even among different age groups. We think that this up-regulation of the osteoclast differentiation pathway is a stable signature of SS inspite of the phenotypic heterogeneity.

## Conclusion

Firstly, systematic analysis of multiple data sets enabled identification of the core set of genes that are consistently up-regulated in SS. Secondly, we applied three different methods (Over-Representation Analysis, Gene Set Enrichment Analysis and Signaling Pathway Impact Analysis) to arrive at the Osteoclast differentiation pathway with consistent and significant up-regulation in SS. We validated this result with additional bioinformatic analysis and pathway gene expression assay in two independent validation data sets. Lastly, we identified 25 genes that may serve as an expression signature of SS. In light of this finding, altered osteoclast differentiation in septic shock deserves greater attention.

## Supporting information

S1 Fig Dot plot of gene expression. This is a dot plot representing log fold change in gene expression for all the filtered and quantilenormalised genes of each study. Green and Red dots represent down-regulated and up-regulated genes respectively. Any up-regulated gene, i.e., with expression higher in SS compared to healthy controls is shown as a red point, and a downregulated gene is shown as a green point. The base line at 0 corresponds to “no change” in gene expression. The 2 dotted lines correspond to the threshold of 2-fold change (i.e., log-fold change of 1) in expression. Any point above the top dotted line represents a gene whose expression level is twice or more in SS compared with healthy controls. Similarly, any point below the bottom dotted line represents a gene whose expression level is at least half (or lower) in SS compared with healthy controls. Note that a change of 2 in the linear scale corresponds to a change of 1 in the log-2 scale. There are far more up-regulated genes (red points) than down-regulated genes (green points). Additionally there are many up-regulated genes with 2-fold or greater change in gene expression. This figure provides compelling visual evidence of high-intensity gene up-regulation in SS.
(TIF)

S2 Fig IPA Network. Network “Infectious Disease, Inflammatory Response, Connective Tissue Disorders”, generated by Ingenuity Pathway Analysis with top 200 up-regulated genes. The nodes in the diagram are color-coded according to the degree of differential expression (higher the up-regulation, more reddish the node). The un-coloured nodes are genes that are not part of the list of 200 genes.
(TIF)

S3 Fig Correction for Batch Effect. ComBat function (of sva R package) was used to remove the batch effect from validation cohort samples as shown in the box plots above.
(TIF)

S4 Fig Highly significant 25 genes. Box plots of the highly significant 25 genes of the pathway hsa04380 up-regulated in SS. Green color corresponds to the control subjects while the red color corresponds to the cases of SS. Gene symbols are shown at the bottom. For each gene, log-intensity of gene expression has been normalized to the median expression of the control group.
(TIF)

S5 Fig Topology of the Pathway. The above figure represents the gene expression pattern of the 25 genes divided into three groups based on location within the pathway: Membrane, Intermediate, Nucleus. The box plot in inset represents mean log-fold change in gene expression of the three groups.
(TIF)

S6 Fig Gene Expression in Survivors and Non-survivors. Log-fold change in gene expression of the 25 genes for different otucome groups. 13 out of 25 genes are associated with higher fold-change compared to non-survivors (p < 0.1; marked as asterisk at the bottom of plot area) For convenience of visualization, all expression values for a gene were shifted by median expression in the control group.
(TIF)

S1 Table Overlapping Sample Matrices. Overlapping sample matrices of SS and Controls in Discovery cohort. The number in each cell represents the samples shared by two studies.
(PDF)

S2 Table Annotation of up-regulated genes. Annotation of top 200 up-regulated genes into associated principal biological functions.
(PDF)

S3 Table Result of Over Representation Analysis. Top 3 enriched KEGG pathway selected after Over Representation Analysis with 200 up-regulated genes.
(PDF)

S4 Table 25 Genes. Annotation of 25 genes of the pathway hsa04380 that are significantly up-regulated in SS.
(PDF)

S1 File PRISMA Check List. The PRISMA 2009 check list.
(PDF)
