# Supplementary figures and images for: Transcriptomic meta-analysis reveals up-regulation of gene expression functional in osteoclast differentiation in human septic shock
Source: PLoS One. 2017 Feb 15;12(2):e0171689. doi: 10.1371/journal.pone.0171689 (PMC5310888; doi:10.1371/journal.pone.0171689)

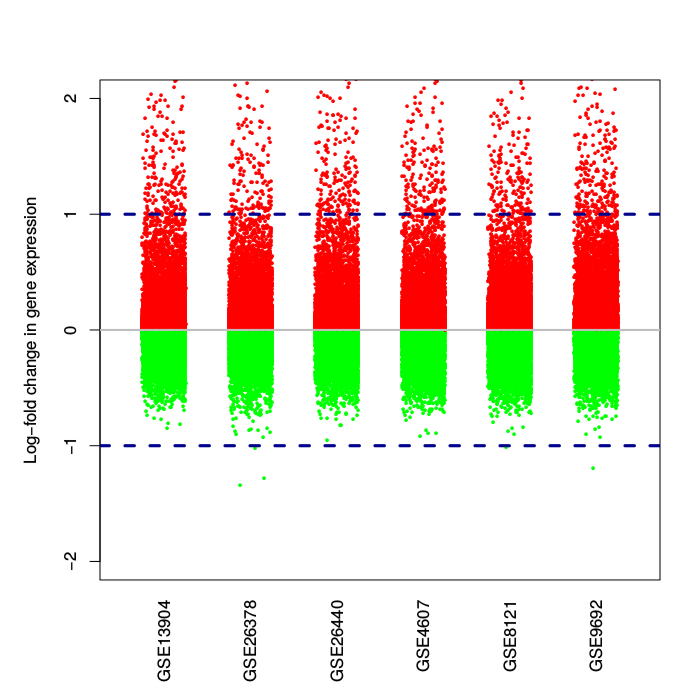

Supplement: S1 Fig — This is a dot plot representing log fold change in gene expression for all the filtered and quantilenormalised genes of each study. Green and Red dots represent down-regulated and up-regulated genes respectively. Any up-regulated gene, i.e., with expression higher in SS compared to healthy controls is shown as a red point, and a downregulated gene is shown as a green point. The base line at 0 corresponds to “no change” in gene expression. The 2 dotted lines correspond to the threshold of 2-fold change (i.e., log-fold change of 1) in expression. Any point above the top dotted line represents a gene whose expression level is twice or more in SS compared with healthy controls. Similarly, any point below the bottom dotted line represents a gene whose expression level is at least half (or lower) in SS compared with healthy controls. Note that a change of 2 in the linear scale corresponds to a change of 1 in the log-2 scale. There are far more up-regulated genes (red points) than down-regulated genes (green points). Additionally there are many up-regulated genes with 2-fold or greater change in gene expression. This figure provides compelling visual evidence of high-intensity gene up-regulation in SS. (TIF) [file pone.0171689.s001.tif]

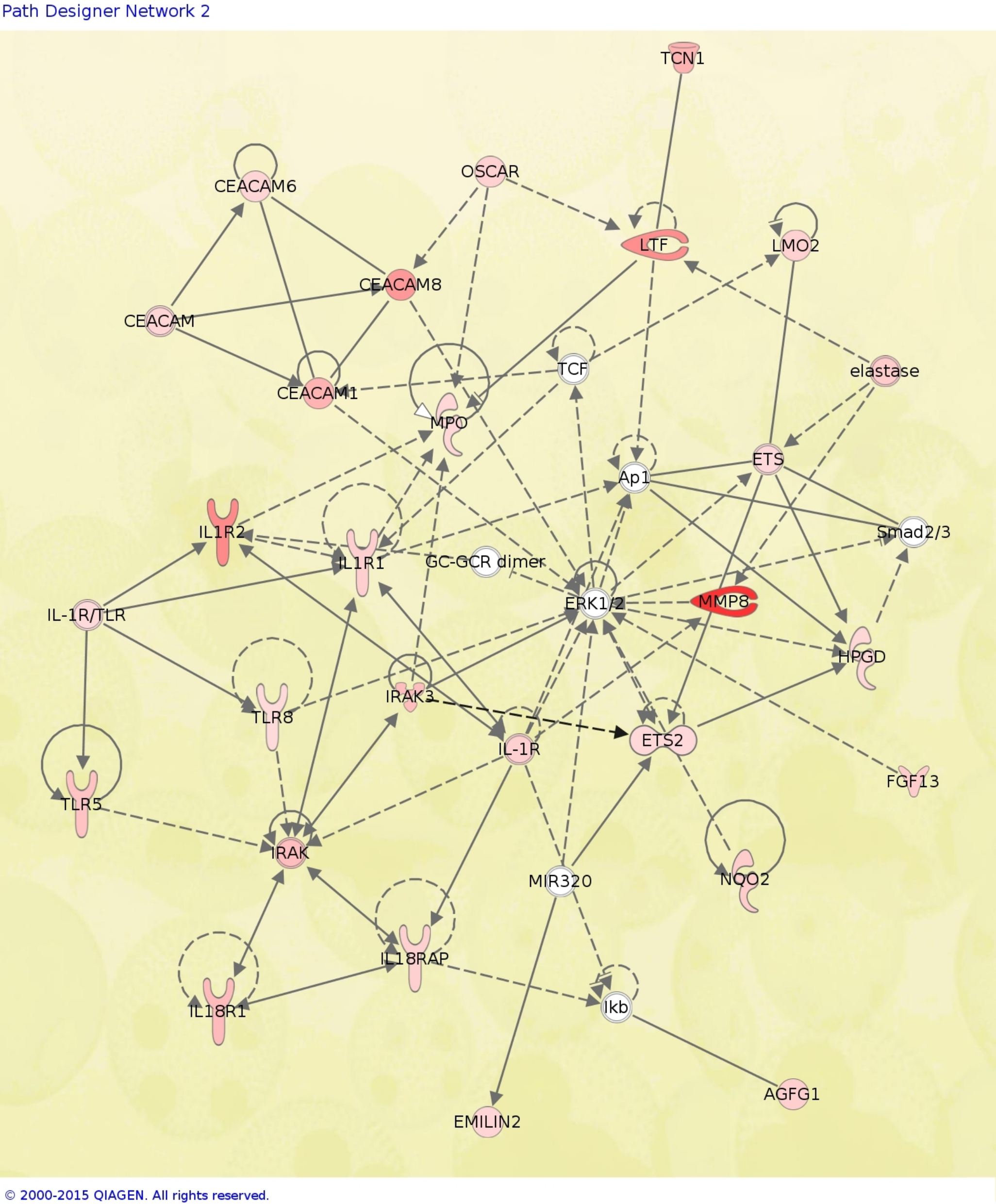

Supplement: S2 Fig — Network “Infectious Disease, Inflammatory Response, Connective Tissue Disorders”, generated by Ingenuity Pathway Analysis with top 200 up-regulated genes. The nodes in the diagram are color-coded according to the degree of differential expression (higher the up-regulation, more reddish the node). The un-coloured nodes are genes that are not part of the list of 200 genes. (TIF) [file pone.0171689.s002.tif]

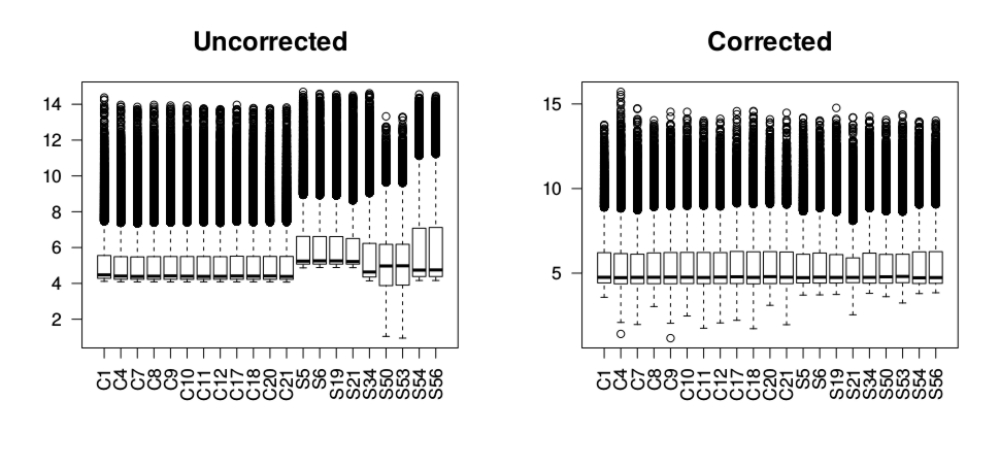

Supplement: S3 Fig — ComBat function (of sva R package) was used to remove the batch effect from validation cohort samples as shown in the box plots above. (TIF) [file pone.0171689.s003.tif]

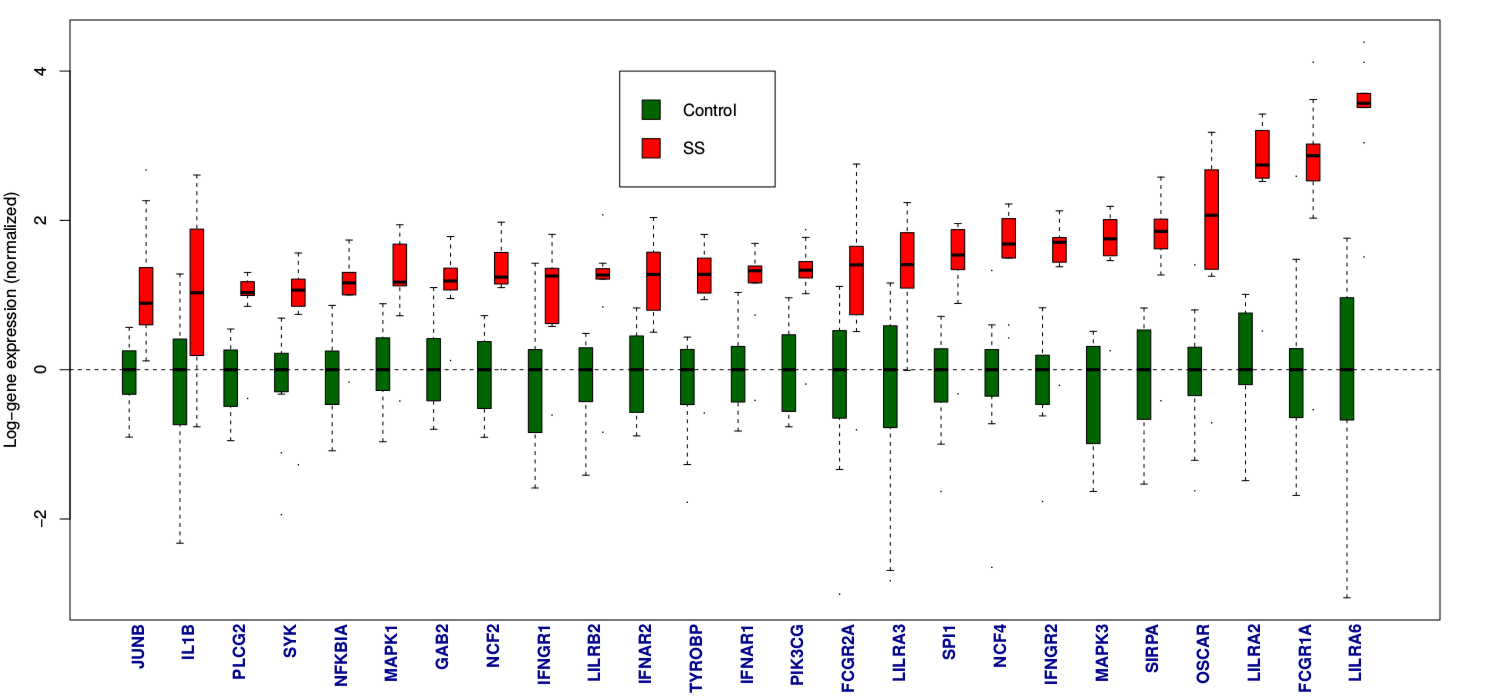

Supplement: S4 Fig — Box plots of the highly significant 25 genes of the pathway hsa04380 up-regulated in SS. Green color corresponds to the control subjects while the red color corresponds to the cases of SS. Gene symbols are shown at the bottom. For each gene, log-intensity of gene expression has been normalized to the median expression of the control group. (TIF) [file pone.0171689.s004.tif]

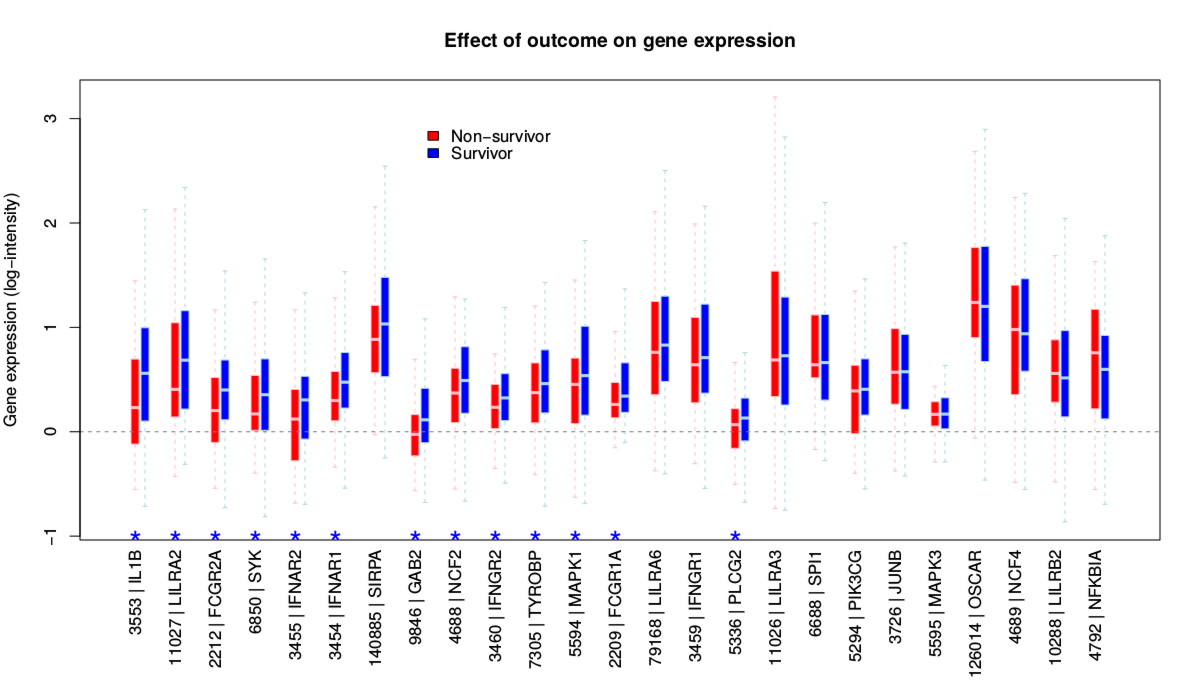

Supplement: S6 Fig — Log-fold change in gene expression of the 25 genes for different otucome groups. 13 out of 25 genes are associated with higher fold-change compared to non-survivors (p < 0.1; marked as asterisk at the bottom of plot area) For convenience of visualization, all expression values for a gene were shifted by median expression in the control group. (TIF) [file pone.0171689.s006.tif]
