# Supplementary material for: Transcriptomic meta-analysis reveals up-regulation of gene expression functional in osteoclast differentiation in human septic shock
Source: PLoS One. 2017 Feb 15;12(2):e0171689. doi: 10.1371/journal.pone.0171689 (PMC5310888; doi:10.1371/journal.pone.0171689)
Supplement: S1 Table — Overlapping sample matrices of SS and Controls in Discovery cohort. The number in each cell represents the samples shared by two studies. (PDF) [file pone.0171689.s007.pdf]

**SS**	**GSE13904**	**GSE26440**	**GSE8121**	**GSE9692**	**GSE4607**	**GSE26378**
**GSE13904**	106	66	0	0	0	0
**GSE26440**	66	98	0	0	0	0
**GSE8121**	0	0	60	30	0	0
**GSE9692**	0	0	30	30	0	0
**GSE4607**	0	0	0	0	69	0
**GSE26378**	0	0	0	0	0	82
**CONTROL**	**GSE13904**	**GSE26440**	**GSE8121**	**GSE9692**	**GSE4607**	**GSE26378**
**GSE13904**	18	18	0	0	0	0
**GSE26440**	18	32	0	0	0	0
**GSE8121**	0	0	15	15	0	0
**GSE9692**	0	0	15	15	0	0
**GSE4607**	0	0	0	0	15	0
**GSE26378**	0	0	0	0	0	21
