# Supplementary material for: Transcriptomic meta-analysis reveals up-regulation of gene expression functional in osteoclast differentiation in human septic shock
Source: PLoS One. 2017 Feb 15;12(2):e0171689. doi: 10.1371/journal.pone.0171689 (PMC5310888; doi:10.1371/journal.pone.0171689)
Supplement: S2 Table — Annotation of top 200 up-regulated genes into associated principal biological functions. (PDF) [file pone.0171689.s008.pdf]

Functional (disease) Module	Genes	Previous Reports where the gene or the module has been reprorted
**Septic Shock**	ANKRD22, CEACAM1, CLEC5A, G0S2, BCL-6, MMP8, TREM1, CCL4	[1,2]
**Inflammation & Innate Immunity**	SERPINB1, S100A12, SLC11A1, TLR5, PADI4, CLEC5A, TLR8, LILRA5	[3, 6, 7, 8]
**Anti Microbial Activity**	ADM, BP1, DEFA4, RNASE3	[3]
**Complement System**	C1QB, F5, CR1, C3AR1	[3, 8, 9]
**Coagulation System**	CR1, C3AR1, CD59, SERPINA1	[3, 8, 9]
**Carbohydrate Metabolism**	PFKB2, PFKB3, SLC2A3, GYG1, PYGL	[4]
**Lipid Metabolism**	ACSL1, HGF, FAR2, DGAT2	[5]
**Bone Metabolism**	CPD, MAPK14, FOSL2 IL1R1, MAP2K6, SOCS3, OSCAR, SIRPA, ALPL, BST1, CA4, TNFAIP6	[3]
**Calcium Signalling**	S100A12, DYSF, MRVI1, LILRA5	[3]
**Cytoskeletal & Cell Adhesion**	LIMk2, DSC2	[3]
**Cancer-associated Genes**	BCL2A1, BCL6, CEACAM1, CD63, DACH1, DSC2, ELANE, ETS2, HPGD, IL1RN, LMO2, MMP8, MMP9, MPO, NQO2, IL1R2, PLXNC1, OLFM4, RAB20	[3]
**MAP kinase genes**	MAPK14, MAP2K6	[6, 9]
