# Supplementary material for: Transcriptomic meta-analysis reveals up-regulation of gene expression functional in osteoclast differentiation in human septic shock
Source: PLoS One. 2017 Feb 15;12(2):e0171689. doi: 10.1371/journal.pone.0171689 (PMC5310888; doi:10.1371/journal.pone.0171689)
Supplement: S3 Table — Top 3 enriched KEGG pathway selected after Over Representation Analysis with 200 up-regulated genes. (PDF) [file pone.0171689.s009.pdf]

KEGG ID	p	Odds Ratio	Pathway Name
**hsa04610**	0.00001	11.5	Complement and coagulation cascades
**hsa04380**	0.00006	6.59	Osteoclast differentiation
**hsa05202**	0.00046	4.77	Transcriptional misregulation in cancer
