# Supplementary material for: Transcriptomic meta-analysis reveals up-regulation of gene expression functional in osteoclast differentiation in human septic shock
Source: PLoS One. 2017 Feb 15;12(2):e0171689. doi: 10.1371/journal.pone.0171689 (PMC5310888; doi:10.1371/journal.pone.0171689)
Supplement: S4 Table — Annotation of 25 genes of the pathway hsa04380 that are significantly up-regulated in SS. (PDF) [file pone.0171689.s010.pdf]

**Entrez Ids**	**Gene Symbols**	**Gene expressed in Tissues**	**Protein levels in plasma**
2209	FCGR1A	NK Cells	Detected
2534	FCGR2A	Monocyte, Whole Blood	Detected
9846	GAB2	CD14+ Monocyte, Whole Blood	Detected
3454	IFNAR1	NK Cells	Detected
3455	IFNAR2	Dendritic Cells	Detected
3459	IFNGR1	Whole Blood, Monocyte	Detected
3460	IFNGR2	NK cells , Monocyte, Whole Blood	Detected
3552	IL1A	Monocyte	Detected
3726	JUNB	Myeloid	Detected
11027	LILRA2	Monocyte	Detected
11026	LILRA3	Monocyte	Detected
79168	LILRA6	Monocyte	Detected
10288	LILRB2	Monocyte	Detected
5594	MAPK1	CD56+ NK cells, Monocyte, Whole Blood	Detected
5595	MAPK3	Whole Blood, Monocyte	Detected
4688	NCF2	Whole Blood, Monocyte	Detected
4689	NCF4	Whole Blood, Monocyte	Detected
4792	NFKBIA	CD33+ Myeloid	Detected
126014	OSCAR	Neutrophil	Detected
5294	PIK3CG	NK cells, Monocyte, Whole Blood	Detected
5336	PLCG2	NK Cells	Detected
140885	SIRPA	Whole Blood	Detected
55423	SYK	Whole Blood, Monocyte	Detected
6688	SPI1	Whole Blood, Monocyte	Detected
7305	TYROBP	Monocyte	Detected
