# Supplementary material for: Transcriptomic meta-analysis reveals up-regulation of gene expression functional in osteoclast differentiation in human septic shock
Source: PLoS One. 2017 Feb 15;12(2):e0171689. doi: 10.1371/journal.pone.0171689 (PMC5310888; doi:10.1371/journal.pone.0171689)
Supplement: S1 File — The PRISMA 2009 check list. (PDF) [file pone.0171689.s011.pdf]

PRISMA 2009 Checklist

Section/topic	#	Checklist item	Reported on page #
**TITLE**			
Title	1	Identify the report as a systematic review, meta-analysis, or both.<br>Transcriptomic Meta-analysis Reveals Up-regulation of Gene<br>Expression Functional In Osteoclast Differentiation in<br>Human Septic Shock<br><br>The report is a meta-analysis.	1
**ABSTRACT**			
Structured summary	2	Provide a structured summary including, as applicable: background; objectives; data sources; study eligibility criteria, participants, and interventions; study appraisal and synthesis methods; results; limitations; conclusions and implications of key findings; systematic review registration number.<br><br>Septic shock is a major medical problem with high morbidity and mortality and incompletely understood biology. Integration of multiple data sets into a single analysis framework empowers discovery of new knowledge about the condition that may have been missed by individual analysis of each of these data sets. Electronic search was performed on medical literature and gene expression databases for selection of transcriptomic studies done in circulating leukocytes from human subjects suffering from septic shock. Gene-level meta-analysis was conducted on the six selected studies to identify the genes consistently differentially expressed in septic shock. This was followed by pathway-level analysis using three different algorithms (ORA, GSEA, SPIA). The identified up-regulated pathway, Osteoclast differentiation pathway (hsa04380) was validated in two independent cohorts. Of the pathway, 25 key genes were selected that serve as an expression signature of Septic Shock.	1
**INTRODUCTION**			
Rationale	3	Describe the rationale for the review in the context of what is already known.<br>Genome-wide expression profiling offers a detailed picture of the condition and enables identification of genes and pathways of diagnostic, prognostic or therapeutic relevance. There have been a number of studies investigating gene expression in sepsis and septic shock leading to very interesting discoveries, such as altered zinc metabolism in sepsis, and clinically relevant grouping of cases of septic shock. The common goal of these previous analyses was to detect interesting genes associated with sepsis or septic shock. Gene-level analysis is inherently not geared toward detection of pathways, which are sometimes modulated even in the absence of significant gene-level changes in expression.	1
Objectives	4	Provide an explicit statement of questions being addressed with reference to participants, interventions, comparisons, outcomes, and study design (PICOS).	

		NA	
**METHODS**			
Protocol and registration	5	Indicate if a review protocol exists, if and where it can be accessed (e.g., Web address), and, if available, provide registration information including registration number.<br>Code and data package are available under the project ssnibmg at https://figshare.com<br><br>Preprint of the manuscript can be found at: http://biorxiv.org/content/early/2016/05/05/051706	4
Eligibility criteria	6	Specify study characteristics (e.g., PICOS, length of follow-up) and report characteristics (e.g., years considered, language, publication status) used as criteria for eligibility, giving rationale.<br>We searched the popular online database PubMed with the search string ("Systemic Inflammatory Response Syndrome"[MeSH] OR "septic shock"[MeSH] OR "Shock, Septic"[MeSH] OR "Endotoxemia"[MeSH]) AND ("gene expression profiling"[MeSH] OR "transcriptome"[MeSH] OR "microarray analysis"[MeSH] OR "Oligonucleotide Array Sequence Analysis"[MeSH]) with "Human" filter. Additionally, we searched the following gene expression databases: (1) National Centre for Biotechnology Information Gene Expression Omnibus (GEO) and (2) European Bioinformatics Institute ArrayExpress.<br>All queries were made on the 3rd January 2017.	2
Information sources	7	Describe all information sources (e.g., databases with dates of coverage, contact with study authors to identify additional studies) in the search and date last searched.<br>(1) National Centre for Biotechnology Information Gene Expression Omnibus (GEO) and (2) European Bioinformatics Institute ArrayExpress	2
Search	8	Present full electronic search strategy for at least one database, including any limits used, such that it could be repeated.<br>Query string ("Systemic Inflammatory Response Syndrome"[MeSH] OR "septic shock"[MeSH] OR "Shock, Septic"[MeSH] OR "Endotoxemia"[MeSH]) AND ("gene expression profiling"[MeSH] OR "transcriptome"[MeSH] OR "microarray analysis"[MeSH] OR "Oligonucleotide	2

		Array Sequence Analysis"[MeSH]).<br>Database: Pubmed<br>Filter: Human<br>Date: 2017/01/03	
Study selection	9	State the process for selecting studies (i.e., screening, eligibility, included in systematic review, and, if applicable, included in the meta-analysis).<br>Selection of studies was based on the organism (human subjects), tissue of origin (circulating leukocytes from whole blood samples) and the platform technology (gene expression microarray). Only data sets published as full reports were selected.	2
Data collection process	10	Describe method of data extraction from reports (e.g., piloted forms, independently, in duplicate) and any processes for obtaining and confirming data from investigators.<br>Normalized gene expression data from the series matrix files were downloaded from GEO.	2
Data items	11	List and define all variables for which data were sought (e.g., PICOS, funding sources) and any assumptions and simplifications made.<br>Only genes common to all studies were included in the analysis.	2
Risk of bias in individual studies	12	Describe methods used for assessing risk of bias of individual studies (including specification of whether this was done at the study or outcome level), and how this information is to be used in any data synthesis.<br>It is to be noted that we did not check for 'publication bias' as this kind of bias is generally meaningful for candidate gene studies, as opposed to omics studies. (It is highly unlikely for a transcriptomic study to not detect any Differentially Expressed gene and remain unpublished). Additionally, had we tried to pool the transcriptomic data, many potential biases would creep in. Therefore, we meta-analyzed the summary t-statistics, allowing us to avoid such biases. Of note, the data within each study was already available as normalized data to remove intra-study biases. While it is still theoretically possible for some genes to behave heterogeneously between studies, it would be biologically rare to occur within the same tissue (whole blood in this case). All the studies chosen had most subjects of Caucasian ethnicity making inter-study heterogeneity in gene-expression less likely to appear. We chose our initial phenotype (Septic Shock) to be as homogenous as possible. This reduced our number of studies for our meta-analysis to only 7. Hence accounting for inter-study heterogeneous behavior of genes (e.g. random effects models) would reduce our statistical power without significant benefits.<br>Since we have started with normalized omics-data, it is expected that there has been correction of bias.	8

Summary measures	13	State the principal summary measures (e.g., risk ratio, difference in means).<br>P-value, log fold change	3
Synthesis of results	14	Describe the methods of handling data and combining results of studies, if done, including measures of consistency (e.g., I^2) for each meta-analysis.<br>For all genes within each study, we applied a Welch's 2-sample t-test to compare expression values in samples of SS with that in samples of control subjects. A single overall p-value was then computed for each gene by meta-analyzing individual study level t-statistics, as described below. We first transformed each individual t-statistic to a Z-score Z_i retaining its sign by using the quantile transformation. We then followed a fixed effects meta-analysis approach, where the Z-scores are combined using an optimal linear combination with weights equal to square root of the effective sample size of each study (i.e., the harmonic mean of case and control sample sizes). Since some of the studies have overlapping cases and controls as shown in Supplementary Table S1, we derived the correlation matrix among the Z-scores of the 6 studies using a previously known correlation formula for case-control studies . The variance of the linear combination is thus derived as the sum of variances of the numerator terms added to their pairwise covariances based on the inter-study correlations R_ij . This gave the combined meta-analyzed Z-score Z_meta .	3

Section/topic	#	Checklist item	Reported on page #
Risk of bias across studies	15	Specify any assessment of risk of bias that may affect the cumulative evidence (e.g., publication bias, selective reporting within studies).<br>Since we have started with normalized omics-data, it is expected that there has been correction of bias.<br>It is to be noted that we did not check for 'publication bias' as this kind of bias is generally meaningful for candidate gene studies, as opposed to omics studies. (It is highly unlikely for a transcriptomic study to not detect any Differentially Expressed gene and remain unpublished). Additionally, had we tried to pool the transcriptomic data, many potential biases would creep in. Therefore, we meta-analyzed the summary t-statistics, allowing us to avoid such biases. Of note, the data within each study was already available as normalized data to remove intra-study biases. While it is still theoretically possible for some genes to behave heterogeneously between studies, it would be biologically rare to occur within the same tissue (whole blood in this case). All the studies chosen had most subjects of Caucasian ethnicity making inter-study heterogeneity in gene-expression less likely to appear. We chose our initial phenotype	8

<table>
<tr><td></td><td></td><td>(Septic Shock) to be as homogenous as possible. This reduced our number of studies for our meta-analysis to only 7. Hence accounting for inter-study heterogeneous behavior of genes (e.g. random effects models) would reduce our statistical power without significant benefits.
Since we have started with normalized omics-data, it is expected that there has been correction of bias.</td><td></td></tr>
<tr><td>Additional analyses</td><td>16</td><td>Describe methods of additional analyses (e.g., sensitivity or subgroup analyses, meta-regression), if done, indicating which were pre-specified.</td><td></td></tr>
<tr><td colspan="3">RESULTS</td><td></td></tr>
<tr><td>Study selection</td><td>17</td><td>Give numbers of studies screened, assessed for eligibility, and included in the review, with reasons for exclusions at each stage, ideally with a flow diagram.
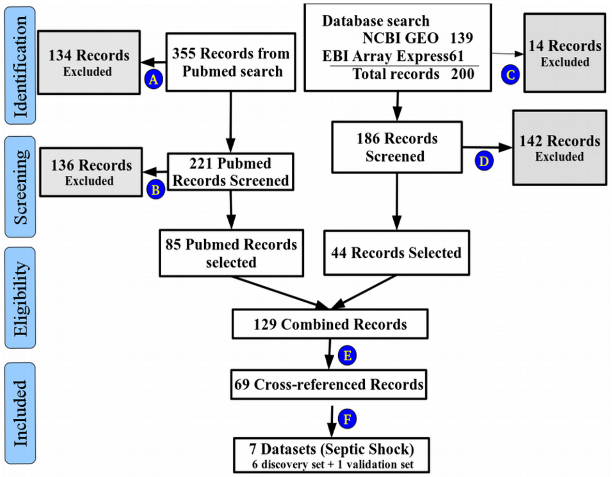
</td><td>Figure 1</td></tr>
<tr><td>Study characteristics</td><td>18</td><td>For each study, present characteristics for which data were extracted (e.g., study size, PICOS, follow-up period) and provide the citations.
PMIDS of the studies included:
<table>
<tr><td>GSE IDs</td><td>PMIDs</td></tr>
<tr><td>GSE4607</td><td>17374846, 18460642, 23107287</td></tr>
<tr><td>GSE8121</td><td>17932561</td></tr>
<tr><td>GSE9692</td><td>18460642</td></tr>
</table></td><td>Table 1</td></tr>
</table>

		GSE13904 19325468<br>GSE26378 21738952<br>GSE26440 19624809<br>GSE57065 26215705	
Risk of bias within studies	19	Present data on risk of bias of each study and, if available, any outcome level assessment (see item 12). We have conducted on omics-data that have already been normalized (with internal correction for bias).	8
Results of individual studies	20	For all outcomes considered (benefits or harms), present, for each study: (a) simple summary data for each intervention group (b) effect estimates and confidence intervals, ideally with a forest plot. The goal of the study was to detect pathways that are consistently up-regulated in septic shock.	6
Synthesis of results	21	Present results of each meta-analysis done, including confidence intervals and measures of consistency. Boxplots of select genes are also provided.	S4 and S6 Figs
Risk of bias across studies	22	Present results of any assessment of risk of bias across studies (see Item 15). Since we have started with normalized omics-data, it is expected that there has been correction of bias.	8
Additional analysis	23	Give results of additional analyses, if done (e.g., sensitivity or subgroup analyses, meta-regression [see Item 16]).	
**DISCUSSION**			
Summary of evidence	24	Summarize the main findings including the strength of evidence for each main outcome; consider their relevance to key groups (e.g., healthcare providers, users, and policy makers).<br>Firstly, systematic analysis of multiple data sets enabled identification of the core set of genes that are consistently up-regulated in SS. Secondly, we applied three different methods (Over-Representation Analysis, Gene Set Enrichment Analysis and Signaling Pathway Impact Analysis) to arrive at the Osteoclast differentiation pathway with consistent and significant up-regulation in SS. We validated this result with additional bioinformatic analysis and pathway gene expression assay in two independent validation data sets. Lastly, we identified 25 genes that may serve as an expression signature of SS. In light of this finding, altered osteoclast differentiation in septic shock deserves greater attention.	9
Limitations	25	Discuss limitations at study and outcome level (e.g., risk of bias), and at review-level (e.g., incomplete retrieval of identified research, reporting bias).<br>This study has a limitation in terms of heterogeneous phenotypes between the discovery and validation cohorts, such as age, and organ involvement. SS is a severe form of disease where the multiorgan involvment and host response is usually similar	8

		even among different age groups. We think that this up-regulation of the osteoclast differentiation pathway is a stable signature of SS inspite of the phenotypic heterogeneity.	
Conclusions	26	Provide a general interpretation of the results in the context of other evidence, and implications for future research. We have identified a set of genes whose expression in septic shock may serve as a signature. In light of this finding, altered osteoclast differentiation in septic shock deserves greater attention.	8
**FUNDING**			
Funding	27	Describe sources of funding for the systematic review and other support (e.g., supply of data); role of funders for the systematic review. NA	

From: Moher D, Liberati A, Tetzlaff J, Altman DG, The PRISMA Group (2009). Preferred Reporting Items for Systematic Reviews and Meta-Analyses: The PRISMA Statement. PLoS Med 6(7): e1000097. doi:10.1371/journal.pmed1000097

For more information, visit: **www.prisma-statement.org**.
